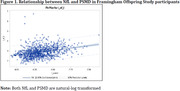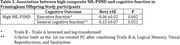# Joint Contribution of NfL and PSMD for Improved Risk Stratification of VCID

**DOI:** 10.1002/alz.093907

**Published:** 2025-01-09

**Authors:** Alison M. Luckey, Alexa S Beiser, Jayandra J. Himali, Carlos A. Gaona, Joy Zeynoun, Luis A. Mendez Rodriguez, Mohamad Habes, Tiffany F. Kautz, Sudha Seshadri, Hugo J. Aparicio, Claudia L Satizabal

**Affiliations:** ^1^ Glenn Biggs Institute for Alzheimer’s & Neurodegenerative Diseases, University of Texas Health Science Center, San Antonio, TX USA; ^2^ Boston University School of Public Health, Boston, MA USA; ^3^ Glenn Biggs Institute for Alzheimer’s & Neurodegenerative Diseases, University of Texas Health Sciences Center at San Antonio, San Antonio, TX USA; ^4^ Boston University Chobanian & Avedisian School of Medicine, Boston, MA USA

## Abstract

**Background:**

Advancing therapeutic and prevention strategies for vascular contributions to cognitive impairment and dementia (VCID) warrants identifying novel biomarkers. However, due to the high heterogeneity underlying dementia pathology, a single marker may not fully risk‐stratify for VCID. A blood‐based biomarker of neuroaxonal injury, neurofilament light chain (NfL), and a neuroimaging‐based biomarker of white matter microstructural damage on diffusion weighted imaging, peak width of skeletonized mean diffusivity (PSMD), have been related to worse general cognition and proposed as robust biomarkers for cerebral small vessel disease (cSVD). We investigated the joint contribution of NfL and PSMD for improved specificity/sensitivity to identify persons at risk for VCID.

**Method:**

Dementia‐free participants from the Framingham Offspring Study with cognitive, neuroimaging, and NfL data were included (N = 969). NfL was measured in plasma, and PSMD was derived from MRI diffusion weighted imaging. Executive function and general cognitive function were assessed from a neuropsychological battery. NfL and PSMD were dichotomized by the top quartile and combined to indicate a high cSVD burden. The high NfL‐PSMD risk category was related to cognitive function using linear regression adjusting for age, age‐squared, sex, education, renal function (eGFR), and total intracranial volume. Additional analyses incorporating amyloid PET uptake in 64 participants were performed to discern Alzheimer’s disease pathology from VCID.

**Result:**

Higher NfL‐PSMD was significantly associated with worse executive (Beta±SE, ‐0.06±0.02, p = 0.002) and general cognitive function (‐0.15±0.07, p = 0.02). Additionally, amyloid PET over‐predicted NfL levels in those with PSMD below the median (observedpredicted, 0.06 (0.33)). Although results were not significant (p = 0.15) due to the limited sample, they suggest that amyloid pathology does not explain cSVD burden measured with NfL and PSMD.

**Conclusion:**

Our findings suggest combining NfL and PSMD can better risk‐stratify those with VCID and poorer cognitive function. This NfL‐PSMD multi‐biomarker has potential to discriminate vascular dysfunction from Alzheimer’s pathology, improving identification of persons better suited for VCID clinical trials. Utilizing a multi‐biomarker approach may improve accuracy for risk stratification of persons bearing covert cerebral vascular injury. Further studies are underway to confirm these findings in larger, diverse samples.